# Sex-specific metabolic interactions between liver and adipose tissue in MCD diet-induced non-alcoholic fatty liver disease

**DOI:** 10.18632/oncotarget.10506

**Published:** 2016-07-09

**Authors:** Yun-Hee Lee, Sou Hyun Kim, Sang-Nam Kim, Hyun-Jung Kwon, Jeong-Dong Kim, Ji Youn Oh, Young-Suk Jung

**Affiliations:** ^1^ College of Pharmacy, Yonsei University, Incheon, Republic of Korea; ^2^ College of Pharmacy, Pusan National University, Busan, Republic of Korea

**Keywords:** sex difference, fatty liver, adipose tissue, lipolysis, FGF21, Pathology Section

## Abstract

Higher susceptibility to metabolic disease in male exemplifies the importance of sexual dimorphism in pathogenesis. We hypothesized that the higher incidence of non-alcoholic fatty liver disease in males involves sex-specific metabolic interactions between liver and adipose tissue. In the present study, we used a methionine-choline deficient (MCD) diet-induced fatty liver mouse model to investigate sex differences in the metabolic response of the liver and adipose tissue. After 2 weeks on an MCD-diet, fatty liver was induced in a sex-specific manner, affecting male mice more severely than females. The MCD-diet increased lipolytic enzymes in the gonadal white adipose tissue (gWAT) of male mice, whereas it increased expression of uncoupling protein 1 and other brown adipocyte markers in the gWAT of female mice. Moreover, gWAT from female mice demonstrated higher levels of oxygen consumption and mitochondrial content compared to gWAT from male mice. FGF21 expression was increased in liver tissue by the MCD diet, and the degree of upregulation was significantly higher in the livers of female mice. The endocrine effect of FGF21 was responsible, in part, for the sex-specific browning of gonadal white adipose tissue. Collectively, these data demonstrated that distinctively female-specific browning of white adipose tissue aids in protecting female mice against MCD diet-induced fatty liver disease.

## INTRODUCTION

Non-alcoholic fatty liver disease (NAFLD) is considered to be one of the most common causes of chronic liver disease and is characterized by fat accumulation without significant alcohol consumption [1]. Although a number of hypotheses have been proposed to help explain the progression of NAFLD, a two-hit model has been the most widely accepted [2–4]. The ‘first hit’ occurs when hepatic triglycerides (TG) accumulate, resulting in fatty liver and increasing the sensitivity of the liver to the ‘secondary hits’, such as inflammatory cytokines/adipokines, mitochondrial dysfunction, and oxidative stress. Following secondary hits, the disease progresses to the next stages-steatohepatitis and hepatic fibrosis.

Methionine-choline deficient (MCD) diet, a well-established experimental model of NAFLD in rodents, rapidly produces the clinical pathologies including macrovesicular steatosis, hepatocyte ballooning, focal inflammation, hepatic necrosis, and fibrosis [5]. Although obesity and insulin resistance are not induced in this model, close similar features found in human NAFLD give the benefit of mechanism study. Moreover, MCD diet reflects a two-hit model very well. Choline deficiency causes decreased synthesis of phosphatidylcholine which is required for very low-density lipoprotein (VLDL), followed by lipid accumulation in the liver [6]. Methionine deficiency induces oxidative stress and inflammation by decreased generation of S-adenosylmethionine and glutathione [7]. Excessive lipid accumulation in the liver is mostly attributable to enhanced uptake and synthesis of fatty acids and to decreased oxidation of fatty acids [8]. Notably, it has been suggested that an inter-organ mechanism contributes to NAFLD, since a decreased capacity for lipid storage alongside increased lipolysis in adipose tissue trigger ectopic lipid accumulation and lipotoxicity in the livers of MCD diet model mice [9, 10].

Adipose tissue can be sub-categorized into brown and white adipose tissue. Brown adipose tissue is a specialized thermogenic organ that can generate heat to maintain body temperature [11]. While white adipose tissue mainly stores or mobilizes lipids as an energy source for systemic use, brown adipose tissue consumes free fatty acids for thermogenesis and mitochondrial oxidation [11]. Importantly, white adipose tissue can adopt the phenotype of brown adipose tissue following exposure to thermogenic stimuli, such as a cold environment, or β-adrenergic stimulation [12]. Thus, the activation and recruitment of the catabolic functions of brown adipocytes has drawn attention as a potential route to promote energy expenditure, and ultimately combat obesity [13]. Moreover, several thermogenic stimuli, including cold stress, can normalize dyslipidemia [14] and improve insulin sensitivity in animal models [15], suggesting that increases in the mass and metabolic activity of brown adipocytes in both brown and white adipose tissue may provide a protective mechanism against obesity-related metabolic disorders [13, 16].

Interestingly, both epidemiological data and animal studies have shown significant sex-specific differences in the prevalence of NAFLD [17–20]. Before the age of 50, men have an increased risk of developing severe NAFLD. In contrast, the incidence of NAFLD after that age is higher in menopausal women than in men, which has suggested the idea that sex hormones might influence the sex-specific onset of NAFLD [18, 20, 21]. In spite of this, some animal studies gave inconsistent results, and so it remains unclear what the basis of the observed sex differences are in NAFLD [22–27]. In the present study, we hypothesized that sex differences in the incidence of NAFLD involve sex-specific metabolic interactions between liver and adipose tissue. We used a liver steatosis model, induced by MCD diet, and investigated sex differences in the metabolic response of liver and adipose tissue.

We demonstrated that 2 weeks on an MCD diet induced liver steatosis prominent in male mice than female in a sex-specific manner. The MCD diet increased the level of lipolytic enzymes in gonadal white adipose tissue (gWAT) of male mice. In contrast, the MCD diet increased the expression of brown adipocyte markers and genes involved in mitochondrial free fatty acid β-oxidation in gWAT of female mice. The MCD diet upregulated FGF21 expression in the liver tissue of female mice, which may explain some mechanisms of the sex-specific effect of the MCD diet on the browning of white adipose tissue. Collectively, these data demonstrated sex-specific induction of browning of white adipose tissue and identified FGF21 as a molecular player in the female specific inter-organ response for protection against MCD-diet induced hepatotocixity.

## RESULTS

### An MCD diet produces male-specific liver toxicity

**Figure 1:**
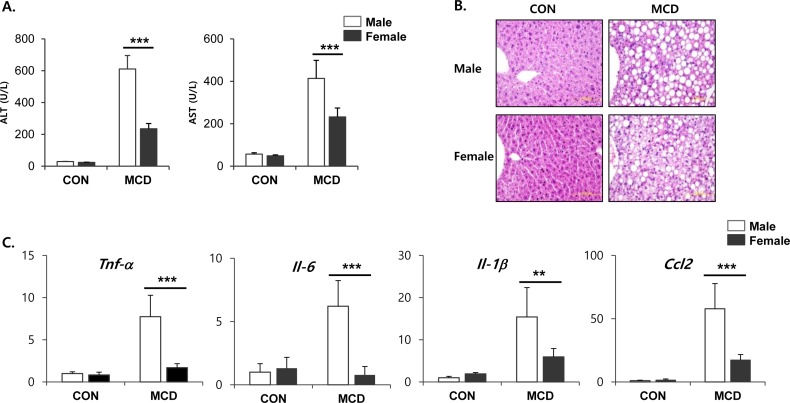
Two weeks of MCD diet induced liver steatosis in male specific manner **A.** Levels of ALT and AST activities in serum from control mice and mice treated with MCD diet for 2 weeks. Two way ANOVA revealed significant main effects of MCD in ALT levels (*p* < 0.001) and in AST levels (*p* < 0.001). **B.** Representative hematoxylin and eosin (H&E) staining of paraffin section of liver. **C.** Quantitative PCR analysis of inflammatory gene expression in liver from control mice and mice treated with MCD diet for 2 weeks. Two way ANOVA revealed significant main effects of MCD in *Tnf-α* (*p* < 0.01), *Il-6* (*p* < 0.05), *Il-1β* (*p* < 0.001), and *Ccl2* (*p* < 0.001). *n* = 5-6 per group. Bars represent mean+SD. Significant differences between male and female were determined by post-hoc pairwise comparison with Bonferroni correction (**p < 0.01, ***p < 0.001).

**Figure 2:**
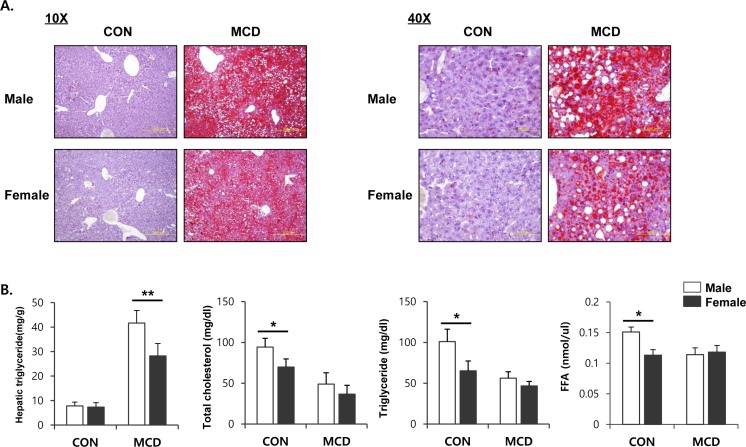
Two weeks of MCD diet increase accumulation of TG in male specific manner **A.** Oil red O staining to determine lipid accumulation in liver (left figure; 10X, right figure; 40X) **B.** Lipid profile in serum and liver. Two way ANOVA revealed significant main effects of MCD in hepatic triglyceride levels (*p* < 0.001), serum total cholesterol (*p* < 0.05), serum triglyceride (*p* < 0.01), and serum FFA (*p* < 0.05). *n* = 5-6 per group. Bars represent mean+SD. Significant differences between male and female were determined by post-hoc pairwise comparison with Bonferroni correction (*p < 0.05, **p < 0.01).

Both male and female mice that had been fed an MCD diet exhibited hepatotoxicity, as evidenced by increased activities of ALT and AST in serum. Interestingly, these levels of enzyme in the serum were higher on this diet in the male mice than in the female mice (Figure 1A). Histopathological analysis showed that the MCD diet induced clear fat vacuoles involving all areas of the hepatic acinus (Figure 1B). We found that steatosis, which was mostly of a macrovesicular type, was more prominent in liver tissue from male mice with weak inflammatory infiltration, than in liver tissue from female mice. It is known that the liver becomes inflamed by an excessive accumulation of lipid, followed by progression to steatohepatitis. In line with this, we determined the expression levels of genes associated with the hepatic inflammatory response. The mRNA expression levels from genes such as *Tnf-α*, *Il-6*, *Il-1β*, and *Ccl2*, which encode inflammatory mediators, was higher in male mice fed an MCD diet compared to similarly fed female mice (Figure 1C). These results correlated with the severity of liver toxicity and steatosis. Staining of neutral triglycerides and lipids (Figure 2A) and the concentration of hepatic triglyceride (Figure 2B) demonstrates that on an MCD diet, hepatic lipid accumulation is higher in male mice than in female mice. While male mice demonstrated lower levels of total cholesterol, triglyceride and free fatty acid levels than female, MCD diet did not affect lipid profiles in serum of male and female mice. (Figure 2B) These results indicate that male mice are more susceptible to the progression of NAFLD than female mice.

### Female mice fed an MCD diet increase their expression of β-oxidation related genes and decrease the expression level of their genes involved in lipid uptake

**Figure 3:**
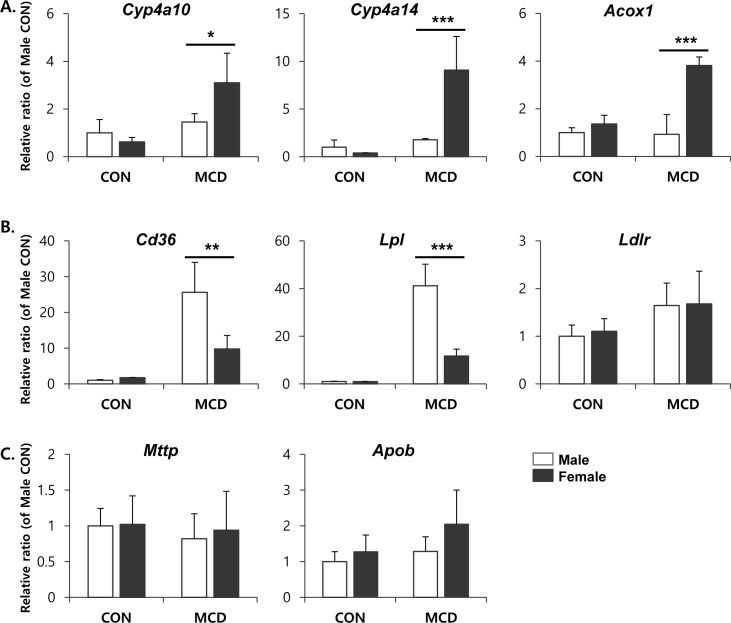
mRNA expression of genes involved in lipid metabolism in liver **A.** fatty acid β-oxidation, **B.** lipid uptake, and **C.** lipid efflux. Two way ANOVA revealed significant main effects of MCD in *Cyp4a10* (*p* < 0.05), *Cyp4a14* (*p* < 0.01), *Acox1* (*p* < 0.01), *Cd36* (*p* < 0.001), and *Lpl* (*p* < 0.001). *n* = 5-6 per group. Bars represent mean+SD. Significant differences between male and female were determined by post-hoc pairwise comparison with Bonferroni correction (*p < 0.05, **p < 0.01, ***p < 0.001).

It has been suggested that an MCD diet promotes lipid accumulation in the liver through increased fatty acid uptake and reduced triglyceride secretion, rather than increased triglyceride synthesis [5]. To understand the sex differences in lipid accumulation in the liver, we focused on the effect of an MCD diet on hepatic expression of the genes involved in fatty acid β-oxidation, as well as lipid uptake and efflux. ACOX1 is the first and rate-limiting enzyme in peroxisomal β-oxidation encoding the enzymes responsible for the transfer shortened fatty acids from peroxisomes to mitochondria [28]. Furthermore, CYP4A genes are coregulated with other genes that encode proteins involved in fatty acid β-oxidation and, functionally, oxidize fatty acids [29]. Therefore, it is considered that CYP4A enzymes are key mediators in hepatic lipid metabolism. The expression levels of genes encoding components of the β-oxidation pathway, including *Cyp4a10*, *Cyp4a14*, and *Acox1*, were significantly higher in female mice on an MCD diet than in similarly fed male mice (Figure 3A). Moreover, compared to male mice, an MCD diet in female mice significantly reduced the expression levels of the genes *Cd36* known as fatty acid translocase and *Lpl* which is a partner of CD36-mediated lipid uptake into liver [30] (Figure 3B). While liver exports lipid in the form of VLDL, which is assembled from triglycerides, cholesterol, and apolipoproteins by microsomal triglyceride transfer protein (MTTP) with apolipoprotein B (ApoB), for utilization by various tissues or storage in the WAT [31]. As shown in Figure 3C, MCD diet didn't affect significantly on the gene expression of lipid export in liver of male as well as female.

### An MCD diet increases lipolysis in the gonadal adipose tissue of male mice

**Figure 4:**
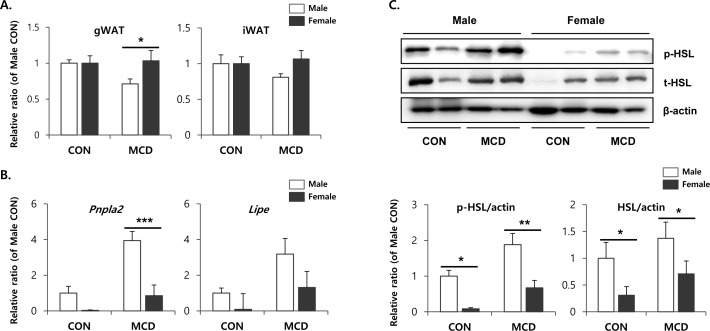
Two weeks of MCD diet increase lipolytic enzyme expression of gonadal white adipose tissue in male specific manner **A.** Measurement of adipose tissue mass of control mice and mice treated with control mice and MCD diet for 2 weeks. **B.** quantitative PCR analysis of genes involved in lipolysis. Two way ANOVA revealed significant main effects of MCD in *Pnpla2* (*p* = 0.01) and *Lipe* (*p* < 0.05). **C.** Western blot analysis and quantification of p-HSL and t-HSL. Two way ANOVA revealed significant main effects of MCD in p-HSL (*p* = 0.003) and t-HSL (*p* < 0.05). *n* = 4 per group. Bars represent mean+SD. Significant differences between male and female were determined by post-hoc pairwise comparison with Bonferroni correction (*p < 0.05, **p < 0.01, ***p < 0.001).

Metabolic interactions between liver and adipose tissue are known to be involved in the pathogenesis of non-alcoholic fatty liver diseases [32]. In particular, hyper-lipolysis of WAT can contribute to TG accumulation in livers in an MCD diet-induced hepatosteatosis model. This observation suggested the possibility that sex differences in the metabolic phenotypes of adipose tissue might confer male-specific hepatotoxic effects during an MCD diet. Therefore, we examined the metabolic phenotype of adipose tissue obtained from male and female mice before and after treatment with an MCD diet. To determine the lipolytic response of various adipose tissue depots following an MCD diet, we examined visceral (i.e., gonadal) and subcutaneous (i.e., inguinal) white adipose tissue, and inter-scapular brown adipose tissue. Among the various adipose tissue depots from distinct anatomic locations, inguinal white adipose tissue (iWAT) is a major subcutaneous adipose tissue depot of mice, and gonadal white adipose tissue (gWAT) is a major dissectible visceral adipose tissue [16]. We chose to monitor these depots, since visceral and subcutaneous adipose tissues have distinct phenotypes, including mechanisms of browning and metabolic responses to lipolytic stimuli [33]. Two weeks on an MCD diet significantly reduced the mass of gWAT in male mice, whereas the same diet had no significant effect on the gWAT mass in female mice (Figure 4A). Similar trends were recorded in the iWAT masses; however, these effects were not statistically significant. Next, we analyzed changes in the expression of lipolytic enzymes at the protein and mRNA levels. Quantitative PCR analysis demonstrated that the expression levels of two genes encoding major lipolytic enzymes, hormone sensitive lipase (HSL) and adipose triglyceride lipase (ATGL), were higher in male mice under basal conditions (Figure 4B). In addition, the expression levels of ATGL and HSL were upregulated in both males and females in response to 2 weeks treatment with an MCD diet. The extent of induction was greatest in the gWAT of male mice (Figure 4B). Consistent with the gene expression analyses, immunoblotting confirmed higher basal and MCD diet-induced expression levels of HSL protein in males compared to females (Figure 4C). Since the metabolic activity of HSL is regulated by protein kinase A (PKA) dependent phosphorylation, we examined the active phosphorylated form of HSL. As expected, levels of phosphorylated-HSL were also increased following 2 weeks on an MCD diet, and the extent of the increase was significantly higher in male gonadal adipose tissue compared to the anatomically similar tissue from female mice (Figure 4C).

### Sex-specific effects of an MCD diet on the browning of gonadal adipose tissue

**Figure 5:**
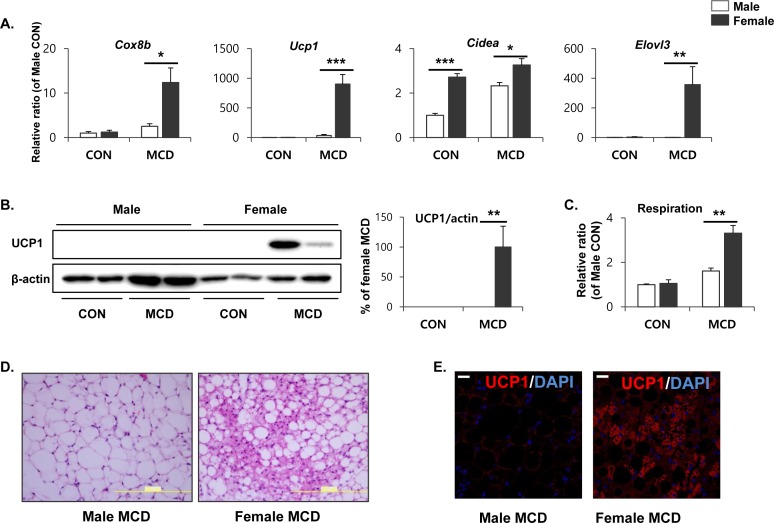
Two weeks of MCD diet increase brown adipocyte marker expression in gonadal white adipose tissue in female specific manner **A.** Quantitative PCR analysis of brown adipocyte markers in gWAT obtained from control mice (CON) and mice treated with MCD diet for 2 weeks. Two way ANOVA revealed significant main effects of MCD in *Cox8b*, *Ucp1*, and *Elovl3* expression (*p* < 0.01). **B.** Western blot analysis and quantification of UCP1 expression in gWAT. Two way ANOVA revealed significant main effects of MCD in UPC1 expression (*p* = 0.001). **C.** Mitochondrial respiration in gWAT as determined by reduction of the electron acceptor dye TTC. Two way ANOVA revealed significant main effects of MCD in respiration (*p* < 0.001). **D.** Representative images of H/E staining of paraffin sections of gWAT (size bars = 200m). **E.** Representative images of UCP1 detection in paraffin sections gWAT (size bars = 20μm). *n* = 4 per group. Bars represent mean+SD. Significant differences between male and female were determined by post-hoc pairwise comparison with Bonferroni correction (*p < 0.05, **p < 0.01, ***p < 0.001).

To determine sex differences in lipid metabolism within adipose tissue, we further examined the expression of genes involved in lipid metabolism within the gonadal adipose tissue of male and female mice. Initially, gene expression profiling was performed by quantitative PCR, and genes involved in mitochondrial β-oxidation and thermogenesis were included. Importantly, cytochrome c oxidase subunit VIIIb (*Cox8b*), a gene that codes for a protein involved in the mitochondrial oxidative metabolism of free fatty acids, was upregulated in female gonadal adipose tissue in response to an MCD diet, indicating that mitochondrial respiration became more active in female gonadal adipose tissue (Figure 5A). Next, we examined the expression of brown adipose marker genes, including a gene encoding the thermogenic protein, uncoupling protein 1 (*Ucp1*). The genes encoding ‘cell death-inducing fragmentation factor alpha subunit-like effector A’ (*Cidea*), and ‘elongation of very long chain fatty acids’ (Elovl3) were upregulated in female gWAT following an MCD diet, compared to male gWAT (Figure 5A). At the protein level, the MCD diet induced UCP1 expression specifically in female gWAT. In contrast, UCP1 protein was undetectable in male gWAT (Figure 5B). The *ex vivo* O_2_ consumption of gWAT, an indicator of mitochondrial respiration, was found to be higher in females when measured by 2,3,5-triphenyltetrazolium chloride (TTC) staining (Figure 5C). Histological analysis of female gWAT established the presence of multilocular (brown) adipocytes (Figure 5D), with intense eosin staining indicating high mitochondrial content. In further support of these data, immunohistochemical analysis demonstrated that UCP1 protein could be detected exclusively in the gWAT from female mice on an MCD diet (Figure 5E). These data suggest that utilization of FFA by mitochondrial respiration becomes more active in female gonadal adipose tissue, compared to the anatomically similar tissue in male mice. These data further support our hypothesis that differences in metabolic phenotypes of white adipose tissue between males and females may contribute to the differential hepatic lipotoxicity, which specifically affects males.

**Figure 6:**
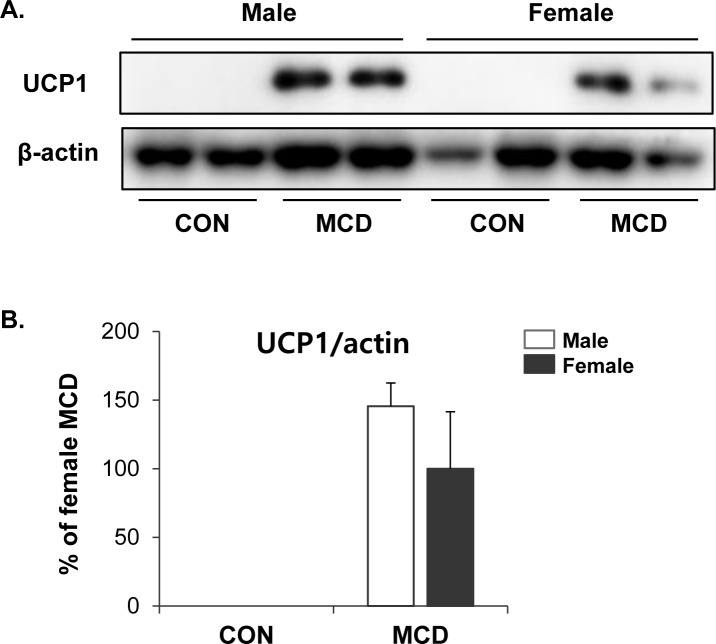
No difference in effects of MCD diet on browning of inguinal white adipose tissue in female and male **A.** Western blot analysis and **B.** quantification of UCP1 expression in iWAT. Two way ANOVA revealed significant main effects of MCD in UCP1 (*p* < 0.01). *n* = 4 per group. Bars represent mean+SD. Significant differences between male and female were determined by post-hoc pairwise comparison with Bonferroni correction.

We further examined the metabolic phenotypes of subcutaneous white adipose tissue following exposure to the MCD diet. Interestingly, no significant differences were detected in the protein levels of UCP1 expression in iWAT between female and male adipose tissues (Figure 6), indicating that there may be no sex differences in the browning of subcutaneous adipose tissue induced by an MCD diet.

### An MCD diet induces female-specific upregulation of FGF21 in liver tissue

**Figure 7:**
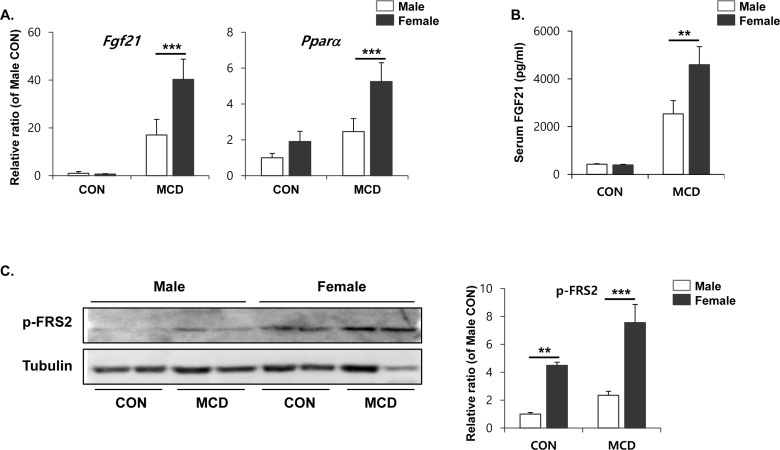
Two weeks of MCD diet induced female specific upregulation of *Fgf21* and *Pparα* expression in liver (**A)** and circulating FGF21 (**B)**, and phospho-FRS2 in gWAT (**C**) Two way ANOVA revealed significant main effects of MCD in hepatic *Fgf21*, (*p* < 0.001), hepatic *Pparα* (*p* < 0.01), circulating FGF21 (*p* < 0.001), and pFRS2 (*p* < 0.01). Bars represent mean+SD. Significant differences between male and female were determined by post-hoc pairwise comparison with Bonferroni correction (**p < 0.01, ***p < 0.001).

We examined expression levels of FGF21, which has been reported to have a brown adipogenic endocrine function [34], in order to determine whether it plays a molecular role in inducing the browning of white adipose tissue. As shown in Figure 7A, FGF21 expression becomes significantly higher in the liver tissue of female mice. Interestingly, PPARα expression, which has been reported as upstream of FGF21, also upregulated more potently in the liver of female mice than male mice (Figure 7A). Consistently, higher levels of FGF21 were detected in serum obtained from female mice compared to that of male (Figure 7B). High levels of FGF21 trigger downstream signaling, including phosphorylation of FRS2. Levels of phosphorylated proteins were detected significantly higher in gonadal adipose tissue from female mice (Figure 7C), suggesting that FGF21 does indeed have an endocrine effect on white adipose tissue in females, but not in the gWAT of males.

**Figure 8:**
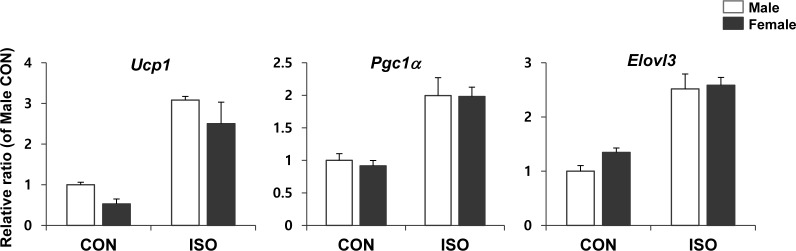
qPCR analysis of brown adipocyte gene expression in differentiated preadipocytes isolated from male and female mice PDGFRα+ cells isolated from white adipose tissue of control mice were exposed to differentiation medium differentiation medium for 4 days and treated with/without 10μM isoproterenol (ISO) for 4 h. Two way ANOVA revealed significant main effects of MCD in *Ucp1* (*p* < 0.001), *Pgc1α* (*p* < 0.01), and *Elovl3* (*p* < 0.001). *n* = 3 per group. Bars represent mean+SD. Significant differences between male and female were determined by post-hoc pairwise comparison with Bonferroni correction.

To determine whether the intrinsic properties of adipocytes from male and female mice are distinct, we tested the brown adipogenic potential of primary cultured adipocytes. Adipocyte progenitor populations from each of the gonadal adipose tissues were isolated and differentiated into adipocytes, using a standard adipogenic cocktail. The expression levels of genes associated with brown adipogenesis were measured by qPCR following isoproterenol treatment. In an *in vitro* model, isoproterenol was equally effective at inducing the browning of primary cultures obtained from both male and female mice (Figure 8), suggesting that the intrinsic ability of adipocytes to transform into brown adipose tissue upon stimulation is similar in both sexes.

## DISCUSSION

The mechanism responsible for the progression of NAFLD in humans is not yet fully understood. Moreover, sex differences in the incidence of NAFLD complicate efforts to understand its pathogenesis. Recently, it has been suggested that the interaction between peripheral tissues, mainly adipose tissue and liver tissue, play an important role in the development of NAFLD [35]. In particular, the increased flux of FFA from lipolysis within adipose tissue contributes to 60% of the hepatic fat content in a fatty liver [2, 36]. Therefore, the importance of adipose tissue in NAFLD is increasing recognized. In this study, we tried to understand sex differences in NAFLD based on metabolic interactions between liver tissue and adipose tissue.

Adipose tissue can store excess energy, and it can limit accumulation of FFA in non-adipose organs. Hyper-lipolysis can cause excessive mobilization of FFA from WAT to the circulation, and consequently to non-adipose tissue. Thus, dysregulation of FFA mobilization can cause aberrant lipid accumulation, leading to lipotoxicity and inflammation. In this study, male mice were more vulnerable to hepatic lipid accumulation while on an MCD diet, compared to female mice. Hepatic lipid metabolism was marked by increased β-oxidation, which uses fatty acids, and decreased lipid uptake in MCD diet fed female mice, compared to male mice. These findings suggest that similar mechanisms may contribute to the sex differences in NAFLD.

Concomitant increases in oxidative metabolism and mitochondrial functioning upon lipolytic signaling is beneficial, since it counterbalances FFA efflux, improving the global metabolic phenotype. Thus, browning of white adipose tissue can increase thermogenesis and FFA catabolism, improving the metabolic profile and reducing hepatotoxicity. In our present study, we have discovered that sex-specific upregulation of in FGF21 expression in mice in response to an MCD diet is correlated with different levels of browning of adipose tissue. This finding may explain, in part, how sex-specific adaptive adipose remodeling aids in protection against the hepatotoxicity induced in mice on an MCD diet.

Sympathetic β-adrenergic stimuli are a major regulatory mechanism that controls lipolysis, mitochondrial β-oxidation of FFA and thermogenic programs in both brown and white adipose tissue. In addition to β-adrenergic stimulation, several non-canonical mechanisms of brown adipocyte activation and recruitment have been identified. Among those, FGF21 is one of the endocrine factors (released mainly from liver) that can act on white adipose tissue to induce browning [34, 37, 38]. Genetic overexpression FGF21 or pharmacological treatment of FGF21 can correct glucose and lipid metabolism [39] and has beneficial effects in mouse models of obesity [38, 40]. FGF21-induced weight loss resulted from increased energy expenditure, in part, if not entirely, by browning of white adipose tissue. Thus, FGF21 is emerging as a promising therapeutic for treating type 2 diabetes and the metabolic syndrome related to obesity [38]. While our study demonstrated that sex-specific upregulation of FGF21 in liver tissue activates a brown adipocyte phenotype in gWAT, the mechanism of sex-specific upregulation of FGF21 has not been identified. We speculate that the molecular mechanism of sex-specific browning of adipose tissue may include effects from sex hormones, such as estrogen. Estrogen can interact with several transcriptional regulators involved in lipid metabolism, including PPARs [41]. It has been demonstrated that FGF21 can be induced by signaling downstream of PPARα in the liver. The potential involvement of sex hormones in the induction of FGF21 deserves further investigation.

In general, levels of browning in white adipose tissue vary, depending on the anatomic location of adipose tissues, the genetic background of individuals, and their sex [42]. For instance, subcutaneous white adipose tissue, the so-called “beige” adipose depot, is more prone to browning upon cold exposure and β-adrenergic stimulation compared to gonadal white adipose tissue [33, 43]. However, mechanisms underlying the differences in browning susceptibility of distinct white adipose tissue depots are not been fully understood [33, 43]. Some evidence suggests that differences in cellular origins and intrinsic properties of adipocytes in various anatomic locations may account for the differing responsiveness to thermogenic stimuli [33, 44]. While sex differences in the browning of white adipose tissue have not been investigated, sex differences in brown adipose tissue have been reported. Brown adipose tissue in female mice upregulates BMP8b, in contrast to that in male mice [45]. It would be informative to investigate which other thermogenic factors are implicated in the sex-specific browning of white adipose tissue.

Inter-organ crosstalk, between the liver and the adipose stores, plays an important role in lipid handling and lies at the base of the pathophysiology associated with the metabolic syndrome, in terms of ectopic lipid deposition and lipid spillover from adipose tissue. Beneficial effects on lipid metabolism arising from the browning of white adipose tissue have been reported for several metabolic diseases. In this study, we have identified the browning of white adipose tissue as a sex-specific protective mechanism against hepatic injury in female mice. Further investigation of the mechanisms of sex-specific upregulation of FGF21 may prove to be useful for the clinical application of FGF21-based therapeutics and may inform preventive approaches for sex-specific diseases of hepatic lipid metabolism.

## MATERIALS AND METHODS

### Animals and diet

All animal protocols were approved by the Institutional Animal Care and Use Committees at Pusan National University (PNU-2015-0957). C57BL/6N mice (6 weeks old, male and female) were purchased from the Orient-Bio (Sungnam, Korea) and acclimated to temperature (22±2°C) and humidity (55±5%) controlled rooms with a 12 h light/dark cycle for 1 week prior to use. 11 mice in each sex were randomly divided into 2 groups and fed a diet deficient in methionine and choline (MCD diet) (*n* = 6) or the control diet with methionine and choline (*n* = 5) for 2 weeks. The diets were obtained from Dyets Inc. (Bethlethem, PA, USA). In the experiment of primary adipocyte culture, 3 male and 3 female mice were used.

### Serum parameters and histological analysis

Serum activity of alanine aminotransferase (ALT) and aspartate aminotransferase (AST) was measured by the protocol of Reitman and Frankel [46]. The levels of triglyceride and free fatty acids were determined enzymatically using a commercially available kit according to the manufacturer's protocol (Sigma Aldrich). Serum level of fibroblast growth factor 21 (FGF21) was measured using the Quantikine ELISA kit (R&D Systems, Minneapolis, MN, USA). For histopathologic evaluation, the liver and adipose tissue were processed, embedded in paraffin, and sliced at 10 μm. TG accumulation was measured by staining with Oil red O (Sigma Aldrich). Sections were counterstained with hematoxylin for 2 min followed by microscopic examination. UCP1 protein was detected in paraffin sections of adipose tissue by standard immunohistochemical (IHC) methods, as previously described [47]. Anti-UCP1 antibody (Alpha Diagnostic International, San Antonio, TX, USA) and anti-rabbit-Alexa Flor-594 (Thermo Scientific, Sunnyvale, CA, USA) were used for IHC detection. Normal rabbit IgG (Santa Cruz, Dallas, TX, USA) was used for negative controls. The sections were counterstained with DAPI (Sigma Aldrich).

### TTC reduction assay

NADH/NADPH dehydrogenase activity of adipose tissue minces *in situ* was detected by measuring the reduction of 2,3,5-triphenyltetrazolium chloride (TTC; Sigma Aldrich, St. Louis, MO, USA), as previously described [48].

### Cell culture

PDGFRα+ cells isolated by magnetic cell sorting (MACS) from white adipose tissue of control mice were cultured to confluence in growth medium (Dulbecco's modified Eagle's medium, DMEM; Welgene, Gyeongsan, South Korea) supplemented with 10% fetal bovine serum (FBS; Thermo Scientific) and 1% penicillin/streptomycin (Thermo Scientific) at 37°C in a humidified atmosphere with 5% CO_2_ and exposed to differentiation medium (DMEM supplemented with 10% FBS, 1% P/S, and 1μg/ml insulin (Sigma Aldrich) or with the addition of isoproterenol (10μM for 4 h).

### Gene expression

RNA was extracted using TRIzol^®^ reagent (Thermo Scientific) and converted into cDNA by using High Capacity cDNA synthesis kit (Applied Biosystems, Waltham, MA, USA). Quantitative real time-polymerase chain reaction (PCR) was performed using SYBR Green Master Mix (Applied Biosystems) and ABI StepOne PLUS (Applied Biosystems) for 45 cycles and the fold change for all the samples was calculated by the comparative cycle-threshold (Ct) method (i.e. 2^−ΔΔCt^ method). 18S ribosomal RNA or peptidylprolyl isomerase A (PPIA) was used as the housekeeping gene for mRNA expression analysis. cDNA was amplified using the primers which were provided in Table 1.

**Table 1:** List of murine primers used for real time RT-PCR

Symbol	Full name	Primer sequence (5′-3′)	
		Forward	Reverse
*Tnf-α*	tumor necrosis factor alpha	GGCCTCTCTACCTTGTTGCC	CAGCCTGGTCACCAAATCAG
*Il-6*	interleukin 6	TTGCCTTCTTGGGACTGATG	CCACGATTTCCCAGAGAACA
*Il-1β*	interleukin 1 beta	TTCACCATGGAATCCGTGTC	GTCTTGGCCGAGGACTAAGG
*Ccl2*	chemokine ligand 2	CCAGCAAGATGATCCCAATG	CTTCTTGGGGTCAGCACAGA
*Cyp4a10*	cytochrome P450, family4, subfamily a. polypeptide 10	AGTGTCTCTGCTCTAAGCC	CCCAAAGAACCAGTGAAAAG
*Cyp4a14*	cytochrome P450, family4, subfamily a. polypeptide 14	TTGCCAGAATGGAGGATAGG	CAGGAAATTCCACTGGCTGT
*Acox1*	acyl-CoA oxidase 1	CCAATCATGCGATAGTCCTGGC	CTTCAGGTAGCCATTATCCATCTC
*Cd36*	Cd36 antigen	CCTTGGCAACCAACCACAAA	ATCCACCAGTTGCTCCACAC
*Lpl*	lipoprotein lipase	ATCGGAGAACTGCTCATGATGA	CGGATCCTCTCGATGACGAA
*Ldlr*	low density lipoprotein receptor	TTCAGTGCCAATCGACTCAC	TGTGACCTTGTGGAACAGGA
*Mttp*	microsomal triglyceride transfer protein	CTCTTGGCAGTGCTTTTTCTCT	GAGCTTGTATAGCCGCTCATT
*Apob*	apolipoprotein B	TTGGCAAACTGCATAGCATCC	TCAAATTGGGACTCTCCTTTAGC
*Pnpla2*	patatin-like phospholipase domain-containing protein 2	ATCTACGGAGCCTCGGCAG	CCACAGTACACCGGGATAAA
*Lipe*	hormone sensitive lipase	GCTGGGCTGTCAAGCACTGT	GTAACTGGGTAGGCTGCCAT
*Cox8b*	cytochrome c oxidase subunit 8b	TGCGAAGTTCACAGTGGTTC	TCAGGGATGTGCAACTTCA
*Ucp1*	uncoupling protein 1	TGGCCTCTCCAGTGGATGTG	CGTGGTCTCCCAGCATAGAAG
*Cidea*	cell death activator CIDE-A	CGGGAATAGCCAGAGTCACC	TGTGCATCGGATGTCGTAGG
*Elovl3*	elongation of very long chain fatty acids protein 3	ACCTACATGAGAACGCGGAA	GTAGATGGCAAAGCACACGG
*Pgc1α*	peroxisome proliferative activated receptor, gamma, coactivator 1 alpha	ACACCTGTGACGCTTTCGCTG	AAGGACACGCTGTCCCATGA
*Pparα*	peroxisome proliferator activated receptor alpha	TGTCGAATATGTGGGGACAA	AATCTTGCAGCTCCGATCAC
*Fgf21*	fibroblast growth factor 21	AGCATACCCCATCCCTGACT	AGACTTTCTGGACTGCGGTG

### Western blot analysis

Protein samples were obtained by lysing the tissues with ice cold RIPA buffer (0.05 M Tris-HCl, pH 7.4, 0.15 M NaCl, 0.25% deoxycholic acid, 1% NP-40, 1 mM EDTA) supplemented with 1 mM phenylmethylsulfonyl fluoride, 2 mM sodium metavanadate, 1 mM sodium fluoride, and protease inhibitor cocktail. The protein concentration in the lysates was determined by the BCA procedure (Thermo scientific). Equal amounts of protein samples were subjected to SDS-PAGE and then transferred onto a polyvinylidene difuoride (PVDF) membrane (Millipore, Billerica, MA, USA). The membrane was blocked with 5% nonfat dry milk in 100 mM Tris-HCl (pH 7.5), 150 mM NaCl, and 0.2% Tween 20 (T-TBS) for 1 h at room temperature. Western blot was performed using primary antibodies against hormone-sensitive lipase (HSL) (Cell Signaling, Danvers, MA, USA), phospho-FSR2 (Cell Signaling), UCP1 (ADI), tubulin (Cell Signaling) and β-actin (Santa Cruz), and secondary anti-mouse or rabbit horse-radish peroxidase antibodies (Cell Signaling) as described previously [47]. The blots were visualized with SuperSignal West Dura Substrate (Thermo Scientific). ImageJ were used for quantitation of blots.

### Statistical analysis

Statistical analyses were performed with GraphPad Prism 5 software (GraphPad Software, La Jolla, CA, USA.). Comparison among groups was performed using two-way ANOVA, with Bonferroni post-tests to determine the relevant *P* values. Data are presented as mean ± SD and considered statistically significant for *p* < 0.05.

## References

[1] Chalasani N, Younossi Z, Lavine JE, Diehl AM, Brunt EM, Cusi K, Charlton M, Sanyal AJ, American Gastroenterological A, American Association for the Study of Liver D and American College of G (2012). The diagnosis and management of non-alcoholic fatty liver disease: practice guideline by the American Gastroenterological Association, American Association for the Study of Liver Diseases, and American College of Gastroenterology. Gastroenterology.

[2] Browning JD, Horton JD (2004). Molecular mediators of hepatic steatosis and liver injury. J Clin Invest.

[3] Cohen JC, Horton JD, Hobbs HH (2011). Human fatty liver disease: old questions and new insights. Science.

[4] Day CP, James OF (1998). Steatohepatitis: a tale of two “hits”?. Gastroenterology.

[5] Rinella ME, Elias MS, Smolak RR, Fu T, Borensztajn J, Green RM (2008). Mechanisms of hepatic steatosis in mice fed a lipogenic methionine choline-deficient diet. J Lipid Res.

[6] Yao ZM, Vance DE (1988). The active synthesis of phosphatidylcholine is required for very low density lipoprotein secretion from rat hepatocytes. J Biol Chem.

[7] Caballero F, Fernandez A, Matias N, Martinez L, Fucho R, Elena M, Caballeria J, Morales A, Fernandez-Checa JC, Garcia-Ruiz C (2010). Specific contribution of methionine and choline in nutritional nonalcoholic steatohepatitis: impact on mitochondrial S-adenosyl-L-methionine and glutathione. J Biol Chem.

[8] Postic C, Girard J (2008). Contribution of de novo fatty acid synthesis to hepatic steatosis and insulin resistance: lessons from genetically engineered mice. J Clin Invest.

[9] Tanaka N, Takahashi S, Fang ZZ, Matsubara T, Krausz KW, Qu A, Gonzalez FJ (2014). Role of white adipose lipolysis in the development of NASH induced by methionine- and choline-deficient diet. Biochim Biophys Acta.

[10] Jha P, Knopf A, Koefeler H, Mueller M, Lackner C, Hoefler G, Claudel T, Trauner M (2014). Role of adipose tissue in methionine-choline-deficient model of non-alcoholic steatohepatitis (NASH). Biochim Biophys Acta.

[11] Cannon B, Nedergaard J (2004). Brown adipose tissue: function and physiological significance. Physiol Rev.

[12] Young P, Arch JRS, Ashwell M (1984). Brown adipose tissue in the parametrial fat pad of the mouse. FEBS Lett.

[13] Kajimura S, Spiegelman Bruce M, Seale P (2015). Brown and Beige Fat: Physiological Roles beyond Heat Generation. Cell Metab.

[14] Bartelt A, Bruns OT, Reimer R, Hohenberg H, Ittrich H, Peldschus K, Kaul MG, Tromsdorf UI, Weller H, Waurisch C, Eychmuller A, Gordts PLSM, Rinninger F (2011). Brown adipose tissue activity controls triglyceride clearance. Nat Med.

[15] Stanford KI, Middelbeek RJW, Townsend KL, An D, Nygaard EB, Hitchcox KM, Markan KR, Nakano K, Hirshman MF, Tseng Y-H, Goodyear LJ (2013). Brown adipose tissue regulates glucose homeostasis and insulin sensitivity. J Clin Invest.

[16] Harms M, Seale P (2013). Brown and beige fat: development, function and therapeutic potential. Nat Med.

[17] Hashimoto E, Tokushige K (2011). Prevalence, gender, ethnic variations, and prognosis of NASH. J Gastroenterol.

[18] Yatsuji S, Hashimoto E, Tobari M, Tokushige K, Shiratori K (2007). Influence of age and gender in Japanese patients with non-alcoholic steatohepatitis. Hepatology research.

[19] Kirsch R, Clarkson V, Shephard EG, Marais DA, Jaffer MA, Woodburne VE, Kirsch RE, Hall Pde L (2003). Rodent nutritional model of non-alcoholic steatohepatitis: species, strain and sex difference studies. J Gastroenterol Hepatol.

[20] Pramfalk C, Pavlides M, Banerjee R, McNeil CA, Neubauer S, Karpe F, Hodson L (2015). Sex-Specific Differences in Hepatic Fat Oxidation and Synthesis May Explain the Higher Propensity for NAFLD in Men. J Clin Endocrinol Metab.

[21] Daryani NE, Daryani NE, Alavian SM, Zare A, Fereshtehnejad SM, Keramati MR, Pashaei MR, Habibollahi P (2010). Non-alcoholic steatohepatitis and influence of age and gender on histopathologic findings. World J Gastroenterol.

[22] Kamada Y, Kiso S, Yoshida Y, Chatani N, Kizu T, Hamano M, Tsubakio M, Takemura T, Ezaki H, Hayashi N, Takehara T (2011). Estrogen deficiency worsens steatohepatitis in mice fed high-fat and high-cholesterol diet. Am J Physiol Gastrointest Liver Physiol.

[23] Kashireddy PR, Rao MS (2004). Sex differences in choline-deficient diet-induced steatohepatitis in mice. Exp Biol Med (Maywood).

[24] Tian GX, Sun Y, Pang CJ, Tan AH, Gao Y, Zhang HY, Yang XB, Li ZX, Mo ZN (2012). Oestradiol is a protective factor for non-alcoholic fatty liver disease in healthy men. Obes Rev.

[25] Zhang H, Liu Y, Wang L, Li Z, Zhang H, Wu J, Rahman N, Guo Y, Li D, Li N, Huhtaniemi I, Tsang SY, Gao GF, Li X (2013). Differential effects of estrogen/androgen on the prevention of nonalcoholic fatty liver disease in the male rat. J Lipid Res.

[26] Spruss A, Henkel J, Kanuri G, Blank D, Puschel GP, Bischoff SC, Bergheim I (2012). Female mice are more susceptible to nonalcoholic fatty liver disease: sex-specific regulation of the hepatic AMP-activated protein kinase-plasminogen activator inhibitor 1 cascade, but not the hepatic endotoxin response. Mol Med.

[27] Xin G, Qin S, Wang S, Wang X, Zhang Y, Wang J (2015). Sex hormone affects the severity of non-alcoholic steatohepatitis through the MyD88-dependent IL-6 signaling pathway. Exp Biol Med (Maywood).

[28] George J, Liddle C (2008). Nonalcoholic fatty liver disease: pathogenesis and potential for nuclear receptors as therapeutic targets. Mol Pharm.

[29] Rao MS, Reddy JK (2001). Peroxisomal beta-oxidation and steatohepatitis. Semin Liver Dis.

[30] Goldberg IJ, Eckel RH, Abumrad NA (2009). Regulation of fatty acid uptake into tissues: lipoprotein lipase- and CD36-mediated pathways. J Lipid Res.

[31] Hussain MM, Shi J, Dreizen P (2003). Microsomal triglyceride transfer protein and its role in apoB-lipoprotein assembly. J Lipid Res.

[32] Fabbrini E, Sullivan S, Klein S (2010). Obesity and nonalcoholic fatty liver disease: biochemical, metabolic, and clinical implications. Hepatology.

[33] Tchkonia T, Thomou T, Zhu Y, Karagiannides I, Pothoulakis C, Jensen Michael D, Kirkland James L (2013). Mechanisms and Metabolic Implications of Regional Differences among Fat Depots. Cell Metab.

[34] Fisher fM, Kleiner S, Douris N, Fox EC, Mepani RJ, Verdeguer F, Wu J, Kharitonenkov A, Flier JS, Maratos-Flier E, Spiegelman BM (2012). FGF21 regulates PGC-1 and browning of white adipose tissues in adaptive thermogenesis. Genes Dev.

[35] Feldstein AE (2010). Novel insights into the pathophysiology of nonalcoholic fatty liver disease. Semin Liver Dis.

[36] Donnelly KL, Smith CI, Schwarzenberg SJ, Jessurun J, Boldt MD, Parks EJ (2005). Sources of fatty acids stored in liver and secreted via lipoproteins in patients with nonalcoholic fatty liver disease. J Clin Invest.

[37] Owen BM, Mangelsdorf DJ, Kliewer SA (2015). Tissue-specific actions of the metabolic hormones FGF15/19 and FGF21. Trends Endocrinol Metab.

[38] Gaich G, Chien Jenny Y, Fu H, Glass Leonard C, Deeg Mark A, Holland William L, Kharitonenkov A, Bumol T, Schilske Holger K, Moller David E (2013). The Effects of LY2405319, an FGF21 Analog, in Obese Human Subjects with Type 2 Diabetes. Cell Metab.

[39] Schlein C, Talukdar S, Heine M, Fischer Alexander W, Krott Lucia M, Nilsson Stefan K, Brenner Martin B, Heeren J, Scheja L (2016). FGF21 Lowers Plasma Triglycerides by Accelerating Lipoprotein Catabolism in White and Brown Adipose Tissues. Cell Metab.

[40] Foltz IN, Hu S, King C, Wu X, Yang C, Wang W, Weiszmann J, Stevens J, Chen JS, Nuanmanee N, Gupte J, Komorowski R, Sekirov L (2012). Treating Diabetes and Obesity with an FGF21-Mimetic Antibody Activating the betaKlotho/FGFR1c Receptor Complex. Sci Transl Med.

[41] D'Eon TM, Souza SC, Aronovitz M, Obin MS, Fried SK, Greenberg AS (2005). Estrogen regulation of adiposity and fuel partitioning. Evidence of genomic and non-genomic regulation of lipogenic and oxidative pathways. J Biol Chem.

[42] Gesta S, Tseng YH, Kahn CR (2007). Developmental origin of fat: tracking obesity to its source. Cell.

[43] Foster DO, Frydman ML (1979). Tissue distribution of cold-induced thermogenesis in conscious warm- or cold-acclimated rats reevaluated from changes in tissue blood flow: the dominant role of brown adipose tissue in the replacement of shivering by nonshivering thermogenesis. Can J Physiol Pharmacol.

[44] Contreras GA, Lee YH, Mottillo EP, Granneman JG (2014). Inducible brown adipocytes in subcutaneous inguinal white fat: the role of continuous sympathetic stimulation. Am J Physiol Endocrinol Metab.

[45] Grefhorst A, van den Beukel JC, van Houten EL, Steenbergen J, Visser JA, Themmen AP (2015). Estrogens increase expression of bone morphogenetic protein 8b in brown adipose tissue of mice. Biol Sex Differ.

[46] Reitman S, Frankel S (1957). A colorimetric method for the determination of serum glutamic oxalacetic and glutamic pyruvic transaminases. Am J Clin Pathol.

[47] Lee YH, Petkova AP, Konkar AA, Granneman JG (2015). Cellular origins of cold-induced brown adipocytes in adult mice. FASEB J.

[48] Granneman JG, Li P, Zhu Z, Lu Y (2005). Metabolic and cellular plasticity in white adipose tissue I: effects of 3-adrenergic receptor activation. Am J Physiol Endocrinol Metab.

